# Antisense Oligodeoxynucleotide Inhibition as an Alternative and Convenient Method for Gene Function Analysis in Pollen Tubes

**DOI:** 10.1371/journal.pone.0059112

**Published:** 2013-03-20

**Authors:** Fanglei Liao, Lu Wang, Li-Bo Yang, Liyao Zhang, Xiongbo Peng, Meng-xiang Sun

**Affiliations:** 1 College of Chemistry and Life Science, Zhejiang Normal University, Jinhua, China; 2 College of Life Sciences, Wuhan University, Wuhan, China; University of Delhi South Campus, India

## Abstract

Antisense oligodeoxynucleotide (A-ODN) inhibition works well in animal cells. However, there have been few successful examples to date of its application in plants, and more specifically whether the technique can be used in pollen tubes as a model of plant cell growth. NtGNL1 plays an important role in pollen tube development and was thus selected as an indicator to assess the biological effects of A-ODN. An A-ODN inhibition technique was used to down-regulate NtGNL1 expression in tobacco pollen tubes and showed that A-ODNs could quickly enter pollen tubes through the thick wall and cell membrane and effectively block NtGNL1 expression. Phenotype analysis revealed that the down-regulation of NtGNL1 by A-ODNs resulted in abnormalities in endocytosis and subsequent vesicle trafficking, similar to the phenotypes of pollen tubes treated with NtGNL1 RNAi. This investigation confirmed that A-ODNs could specifically inhibit target gene expression, and furthermore demonstrated that A-ODN functioned in a concentration- and duration-dependent manner, because A-ODNs could be degraded when incubated with pollen tubes. Thus, the A-ODN technique was successfully used for gene function analysis in pollen tubes and appears to be an alternative and convenient technique when the *in vitro* pollen tube is used as the study model. This technique will greatly facilitate investigations on the molecular mechanism(s) underlying pollen tube growth.

## Introduction

An alternative and emerging technique, antisense oligodeoxynucleotide (A-ODN) inhibition, has been established and used to silence target genes in cancer [1], [2] and animal research [3], [4]. It was shown to be a powerful method for gene function analysis in the medical sciences [5]. Recently, use of a nanoparticle delivery system has helped A-ODN function more effectively [6]. It was proposed that designing 12−25-nucleotide sequences complementary to the mRNA of a target gene would cause RNase H cleavage, inhibiting target gene mRNA transcription [7] or forming a complex to block translation [8] and would be more target-specific, greatly reducing or eliminating off-target effects.

RNA interference (RNAi) is a widely used method for gene silencing. It is particularly useful in species in which the genetic background is not yet well-understood, although it has also attracted criticism because of possible off-target effects [9]–[11]. Compared to RNAi, A-ODN may provide more effective inhibition [12] and the effect of A-ODN is usually faster due to omission of plasmid construction [13]. Furthermore, chemical modifications of A-ODN, such as PS modification (phosphorothioate modification), make its application more stable [14], [15]. Thus, A-ODN inhibition is potentially a powerful technique for gene silencing. In addition, compared to mutation techniques, A-ODN is uniquely advantageous because it is able to transiently downregulate gene expression for the analysis of gene function in specific developmental phases or plant organs. In fact, examples of A-ODN application have been reported in various plants [12], [16]–[19]. However, basic questions such as whether naked or nanoparticle-packed A-ODNs are more effective, or how ODN permeate the plant cell membrane, remains unclear. Recent evidence suggests that A-ODNs enter the cell *via* endocytosis or other vesicle trafficking [20], [21], with evidence of receptor-mediated endocytosis [22]. However, more research is needed to elucidate the mechanism of A-ODN action within the cell to understand the details of how it functions.

The Arf family of guanine-nucleotide-binding (G) proteins and ARF-guanine exchange factors (ARF-GEFs) play crucial roles in vesicle trafficking [23], [24]. Large ARF-GEFs activate ARF-GTP by exchanging GDP for GTP and thus interact with some effectors, regulating diverse events in vesicle trafficking [25]–[29]. We identified *NtGNL1* in tobacco and, using the RNAi technique, confirmed its essential role in pollen tube growth [30], [31]. Cytological observations indicated that the down-regulation of *NtGNL1* resulted in abnormal post-Golgi trafficking [31]. Based on this detailed background, *NtGNL1* could be a useful target gene for evaluating the A-ODN technique in plant cells.

The pollen tube provides an excellent example of polarized cell growth with rapid extension and the processes of vesicle trafficking visible at the tip [32]. Living pollen tubes are convenient for observing endocytosis with FM4-64, a lipophilic probe that fluoresces on binding the plasma membrane [32], [33]. Thus, the *in vitro* growth system of the pollen tube might facilitate research on both A-ODN application in plants and on the molecular mechanism(s) of A-ODN uptake.

Here, we used A-ODN inhibition techniques to down-regulate *NtGNL1* expression in pollen tubes. Our results revealed that A-ODN passes through the pollen tube wall in culture medium and works to suppress *NtGNL1* expression. A-ODN inhibition resulted in similar phenotypes to those observed in RNAi transgenic plants, indicating the A-ODN worked specifically on its intended target. Thus, we established an alternative and convenient experimental system for gene function analysis in pollen tubes, and the technique may facilitate investigations on the molecular mechanism(s) underlying pollen tube growth.

## Results

### A-ODNs Effectively Permeate into Pollen Tubes

**Figure 1:**
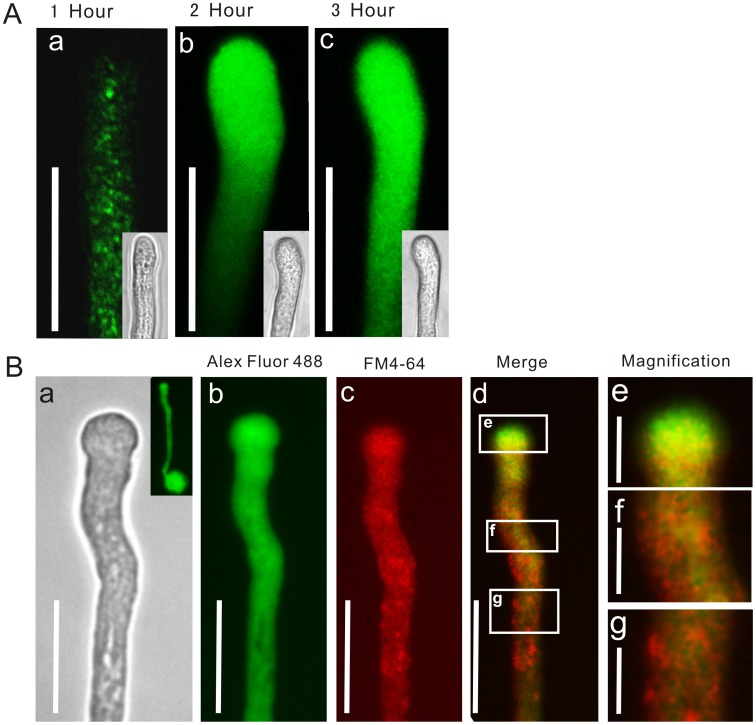
Tracing the uptake of antisense ODN into pollen tubes. A**:** Tracing the uptake of FL-ODNs. FL-ODNs signal appeared as small dots in pollen tubes after 1 hour’s incubation (a). After 2 hours’ incubation, the signal concentrated at the tip of the pollen tube (b) and finally dispersed evenly throughout the entire pollen tube after 3 hour’s incubation (c). a–c Bar = 20 µm. B**:** Pulse-chasing labeling with fluorescence-labeled ODNs and FM4-64. (a) Bright field image of the pollen tube. (b) The image show the same pollen tube labeled with Alexa Fluor 488 fluorescence. (c) The image show the same pollen tube labeled with FM4-64 fluorescence. (d) Pulse-chase labeling with Alexa Fluor 488 for 3 h followed by FM4-64 for 10 min. Squares (e–g) indicate areas magnified in (d), respectively. Bar = 20 µm in (a–d); bar = 10 µm in (e–g). n = 15–20 for the observation of each stages during FL-ODNs uptake.

Unlike animal and plant mesophyll cells, pollen tubes typically have thick cell walls, consisting of esterified homogalacturonan (a major pectin component) at the pollen tube tip, and cellulose and callus in the rigid wall behind the tip [34], [35]. We first tested whether A-ODNs could pass through the pollen tube wall and plasma membrane by labeling a batch of ODNs with Alexa Fluor 488 to monitor the delivery process. Tracing observations revealed that intense Alexa Fluor 488 fluorescence was detectable within pollen tubes after approximately 1 h of incubation (Fig. 1A.a). The fluorescently labeled ODN (FL-ODN) first appeared as small dots or patches in the cytoplasm of the pollen tube (Fig. 1A.a), which then accumulated in the tip region (Fig. 1A.b). After 3 h, the signals had dispersed evenly throughout the pollen tube (Fig. 1A.c). During a 2-h co-culture with FL-ODN, most pollen tubes showed a similar distribution pattern of fluorescent signal (Fig. S1). These results indicate that the ODNs could effectively enter the pollen tubes within a short period.

To determine whether ODN uptake into pollen tubes occurs via endocytosis, we used FM4-64 to track endosome movement after incubation with A-ODNs. Preliminary experiments revealed that the uptake of Alexa Fluor 488 occurs on a much longer time frame than that of FM4-64; thus, we first incubated pollen tubes with FL-ODNs for 2−3 h and then added FM4-64 to visualize endocytosis. Endocytosis occurred primarily in the apex and the shank of the pollen tube. However, the Alex Fluor 488 signal (green) appeared in all parts of pollen tubes, especially at the tip (Fig. 1B.b). The two regions only partially overlapped and displayed distinct distribution patterns, with more green at the apex (Fig. 1B.d) and more red signals along the shank (Fig. 1B.f,g), indicating that the uptake of A-ODN occurred actively at the apex. These results indicate that the A-ODNs likely passed into the pollen tube via some pathway(s) only at the apex.

### A-ODN Specifically Down-regulate *NtGNL1* Expression

**Figure 2:**
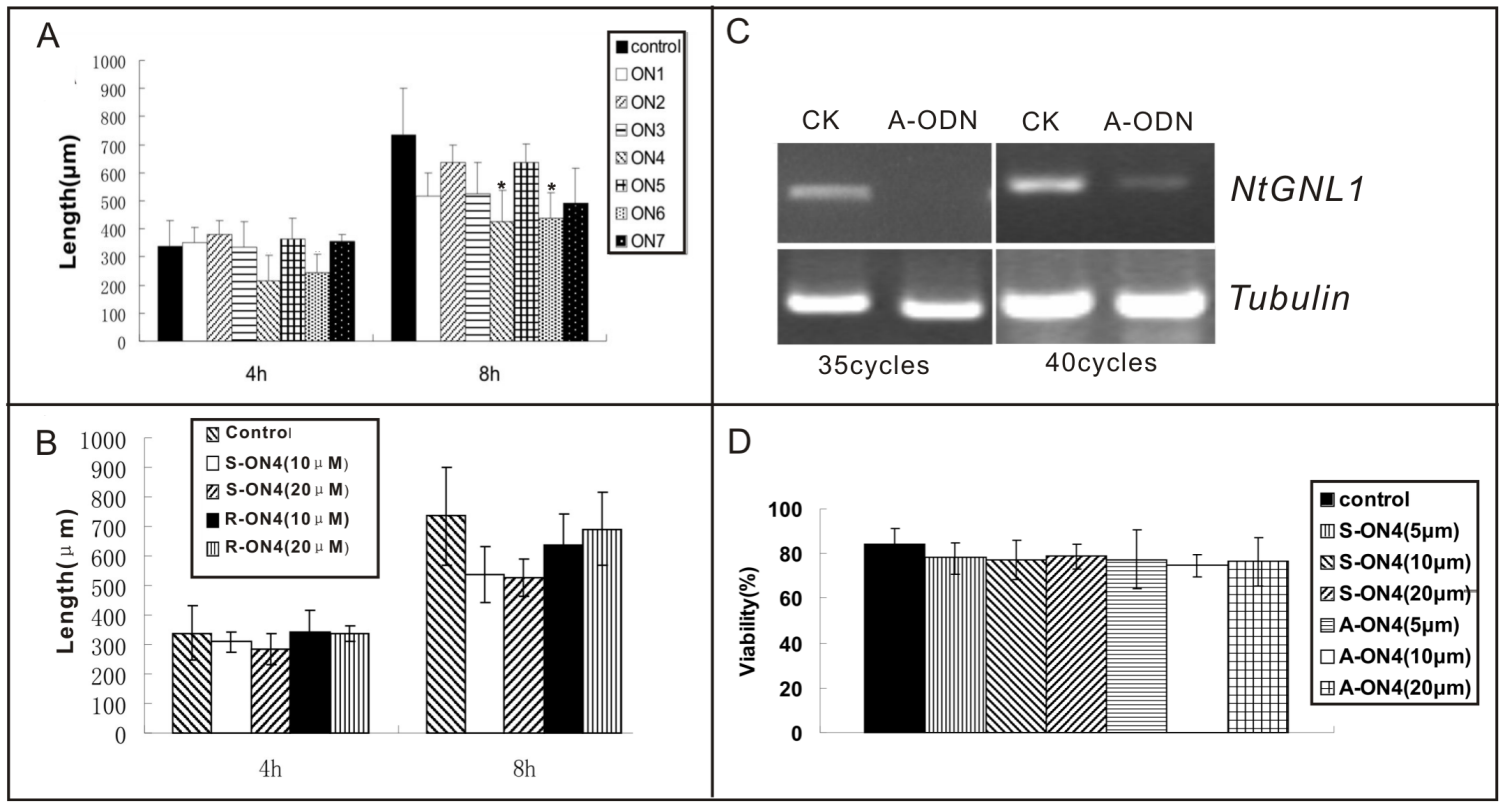
The effect of A-ODNs on pollen tube growth and *NtGNL1* expression level. A: Inhibition effects among antisense ODNs (1.0 µM). n = 300±10. Pollen tubes under ON4 and ON6 treatment were obvious shorter than that under other treatment. Asterisks indicate a significant difference (*P*<0.05). These data were calculated and analyzed by SPSS (16.0) Independent-Sample T Test. Error bars in the columns represent SD. B: No significant inhibition effect on pollen tubes growth was observed during treatment by sense and random ODNs. n = 300±10. C: the effect of antisense ODN treatment on *NtGNL1* mRNA expression. D: the comparison of cytotoxic effect between control (sense or nonsense) and antisense ON4. All of them displayed around 80% viability. n = 300±10.

To evaluate the possibility of off-target and toxic effects of the A-ODN on pollen tube growth, we designed several A-ODNs with differing sequences, based on the *NtGNL1* mRNA sequence (Table 1). We also applied sense ODN and scrambled ODN containing the same nucleotides in a different (nonsense) order (Table 1). These served as controls to observe the pollen tube phenotype. Among these A-ODNs, ON4 and ON6 showed similar effects on pollen tube growth (Fig. 2A). This phenotype was similar to that observed in *NtGNL1* RNAi plants, suggesting the phenotypes involved suppression of NtGNL1 expression. Other ODNs used in these experiments, including the sense ODN and scrambled ODN, showed no significant influence on pollen tube growth (Fig. 2B). To assess *NtGNL1* mRNA expression levels, we extracted RNA from both control and A-ODN-treated pollen tubes, and the results confirmed that *NtGNL1* mRNA expression levels were reduced by the specific A-ODN (Fig. 2C). We primarily used ON4 in the following experiments and selected scrambled (random) sequences of ON4 as a control.

**Table 1:** Sequences and selected positions of antisense ODN.

Name	Position	Sequence(5'–3')
ON1	196	GCTGATTAAGGCACCCCA
ON2	226	CCCTTGGGCTCTGAAATT
ON3	345	CGAAATCCCCACCTCACA
ON4	883	CTGGGCCAGCGCACACTT
ON5	820	CATGCATCGTGTGGCGTG
ON6	998	TCCCCTACGCTCACCAAA
ON7	2149	CGCTTCAAGCACCCTCTG

To test the possible toxic effects of the sequences, we compared the viability of sense- *vs.* ON4-treated pollen tubes (Fig. S2). No significant difference was observed in terms of the percentage of viable pollen tubes from each treatment (Fig. 2D). All treated pollen tubes showed similar viability, suggesting against the possibility of toxic effects due to A-ODN sequences.

### Inhibition of NtGNL1 Expression Disturbs Pollen Tube Elongation and Orientation

**Figure 3:**
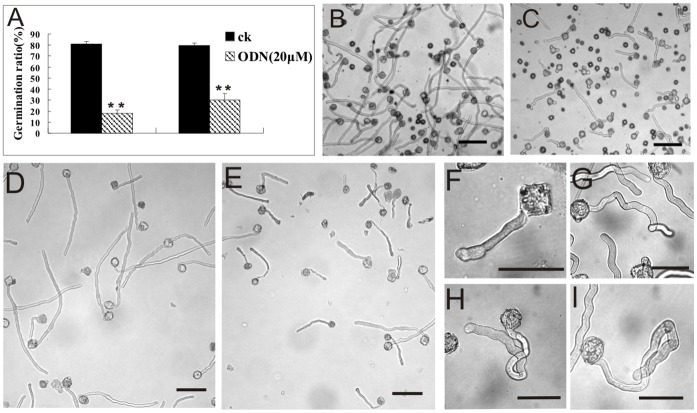
Inhibition effects of A-ODN on pollen germination and pollen tube growth. A, Up to 80% of the control, wild-type pollen germinated after 4 h of culturing, whereas less than 60% of the pollen in antisense ODN media germinated (*n* = 300). Double asterisks indicate P<0.01 between control (ck) and treated sample [SPSS (16.0) Independent-Sample T Test]. Error bars in the columns represent SD. B, A bright field image showing pollen grains germinated in control (CK) after 4 h. C, Approximately half of the pollen grains germinated in A-ODN (20 µM) medium at the same time. Bar = 50 µm. D, E, Pollen tube length comparison between control and A-ODN (5 µM) treated pollen tubes. Pollen tubes in control media (D) were clearly longer than those grown in A-ODN medium (E). Bar = 100 µm. E–I, Phenotypes of A-ODN (20 µM) treated pollen tubes: bulging (F), curved (G), zigzag (H), bending (I).

The germination frequency of ODN-treated pollen was significantly lower than that in the control (Fig. 3A). Indeed, up to 80% of the control pollen germinated within 3 h, whereas only 30% of the potential pollen tubes were observed in the culture media after 4 h of culture with 20 µM ODN (Fig. 3A, C). Although the germination rate may have increased with a longer culture time (data not shown), these data indicated that A-ODN slowed the pollen germination process. The 20-µM A-ODN treatment also significantly disrupted pollen tube growth. Many pollen tubes in the A-ODN medium were much shorter than in the control, as well as in the 5-µM treatment (Fig. 3D, E). ODN-treated pollen tubes grew at a rate of 1.00 µm/min, whereas the control pollen tubes extended by 1.78 µm/min.

Our observations showed that A-ODN treatment also disturbed pollen tube development and orientation. Several abnormal phenotypes were observed in A-ODN-treated pollen tubes, including bulging tips (Fig. 3F), curved tubes (Fig. 3G), zig-zagging (Fig. 3H), and bending at almost a right angle (Fig. 3I). The frequency of bent pollen tubes was 48% among A-ODN-treated tubes (6-h treatment), but only 5.6% in control tubes. Thus, the A-ODN treatment caused clear pollen tube twisting and curling. Similar phenotypes were also observed in RNAi transgenic lines [29], further supporting that the phenotypes observed in A-ODN-treated tubes were the result of the down regulation of the target gene, *NtGNL1*.

### Cytological Phenotypes Observed in ODN-treated Pollen Tubes Were Similar to those in RNAi Lines

**Figure 4:**
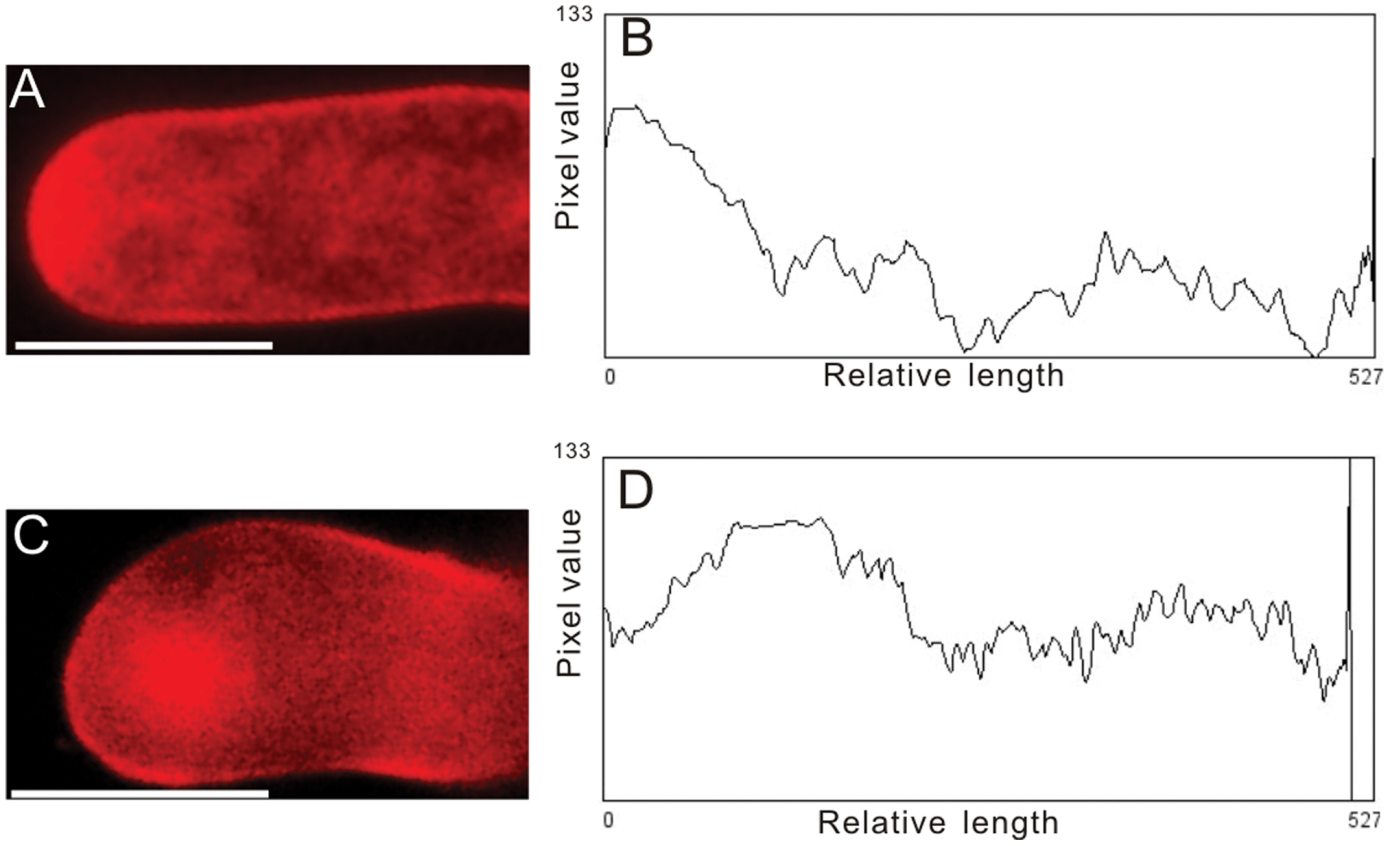
Fluorescent distribution pattern showing uptake FM4-64 within 30 minutes into control and A-ODN treated pollen tubes. A: fluorescent images of a control pollen tube, C: an A-ODN-treated pollen tube. B and D: pixel values (calculated by ImageJ 1.37 v) along a central transect through A and C. Different pixel values curves means different fluorescent distribution pattern. Bar = 20 µm.

To examine the specificity of ODN treatment, we carefully observed the cytological phenotypes in treated pollen tubes. It is known that the down-regulation of *NtGNL1* results in abnormal endocytosis. Following the application of FM4-64, we compared endocytic patterns in control *vs.* A-ODN-treated pollen tubes. Typically, a converse V-shaped distribution was observed in control tubes, whereas A-ODN-treated pollen tubes showed strong fluorescence accumulating in the sub-apical region (Fig. 4), similar to that in RNAi lines [30], [31].

**Figure 5:**
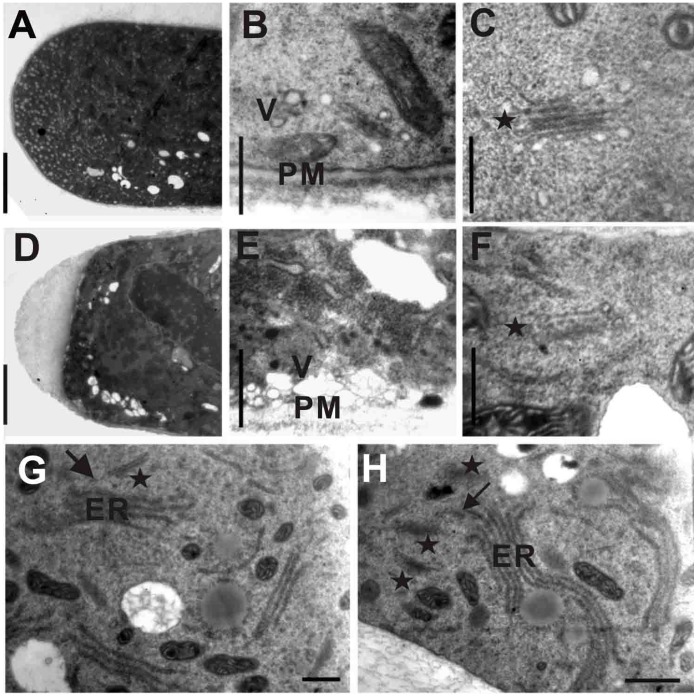
Ultrastructural observation of control and A-ODN treated pollen tubes. A, B, C, G: control, wild-type pollen treated by random ODN; D, E, F, H: A-ODN treated pollen tubes. A, D: Tip region of pollen tubes shows more vesicles at the tip region in control than in ODN-treated pollen tubes, while more and bigger vesicles at the sub-region in the later than the former. Bar = 3 µm. Cell membrane crimpled at the tip of A-ODN treated pollen tubes. B, E: in Sub-apical region of pollen tubes vesicles are more and bigger near the plasma membrane than in control. F: Golgi apparatus disassembled into two cisternae. Bar = 400 µm. PM: plasma membrane; V: vesicle. Pentacles (stars) mean the position of Golgi bodies. G, H: ER extended to Golgi bodied and fused with Golgi bodied in ODN-treated pollen tubes. The arrow heads indicate the fusing ER. Bar = 400 µm.

Ultrastructural observation was also carried out to examine vesicle transport and compartmentalization at the pollen apex and in the sub-apical region. The diameter of the vesicles in control pollen tubes was typically less than 0.2 µm [30], [31]. However, in ODN-treated pollen tubes, more than 10% of the vesicles were larger than 0.4 µm (*n* = 265±10). In addition, larger vesicles were found along the plasma membranes of ODN-treated pollen tubes (Fig. 5A, B, D, and E). The Golgi apparatus also exhibited a variety of abnormalities, as similarly observed in RNAi lines (Fig. 5C, F, G, and H). These results are consistent with those observed in RNAi-treated pollen tubes, suggesting that A-ODN treatment resulted in the same effect as RNAi.

### The Efficacy of A-ODN Treatment Depends on Concentration and Duration

**Figure 6:**
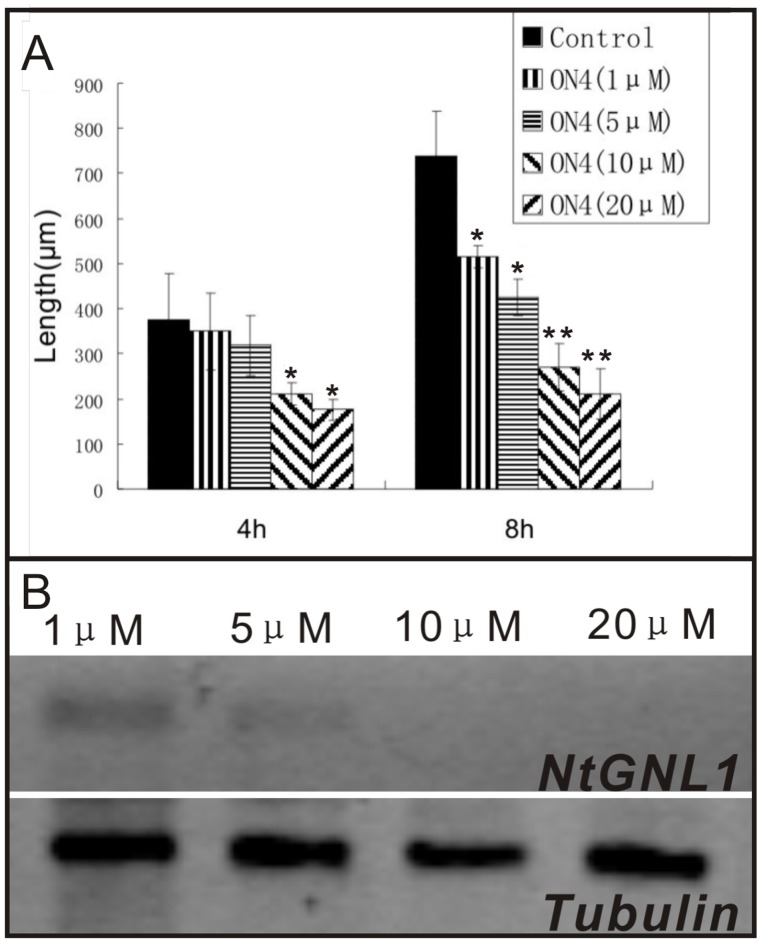
Dosage dependent effect of A-ODN treatment on pollen tube length. A**:** the effect of ODN4 at different concentrations on pollen tubes growth (n = 200±10). Asterisks indicate *P*<0.05, double asterisks indicate P<0.01 between control (ck) and treated sample (SPSS Independent-Sample T Test). Error bars in the columns represent SD. B**:** Dosage dependent effect of A-ODN treatment on *NtGNL1* mRNA expression.

During ODN treatment, we found that pollen tube elongation varied in the presence of various concentrations of ODNs, such that the length of the pollen tube was inversely related to the concentration of ODN (Fig. 6A), as was the mRNA level of *NtGNL1* (Fig. 6B). The mean tube lengths after treatment with 20 µM and 5 µM ODN were approximately 200 µm and 400 µm, respectively. Clearly, higher concentrations of A-ODN were more effective at slowing pollen tube growth, indicating a dose-dependent effect.

**Figure 7:**
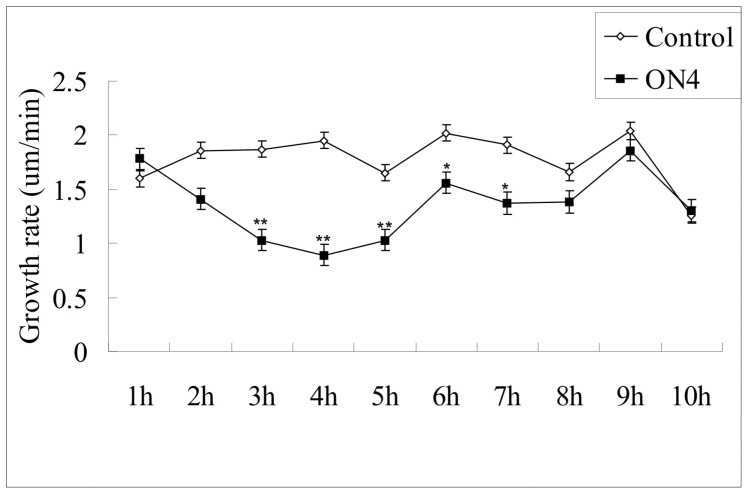
Pollen tube growth rate during A-ODN (20 µM) treatment. The pollen tube growth rate in ODN-treated pollen tubes declined markedly between 3 and 6 h. The double asterisks indicate *P*<0.01, asterisk indicates *P*<0.05. The data were calculated and analyzed by Microsoft Excel 2000 software (Pvalue, student test), *n* = 12±2. Error bars in the columns represent SD.

The efficacy of ODN treatment was also related to the duration of treatment. Notably, the tobacco pollen growth rate during A-ODN treatment declined markedly between 2 h and 6 h (Fig. 7). However, after 8 h of growth, no significant difference was observed between the A-ODN-treated and control pollen tubes, indicating that A-ODN gradually lost its inhibitory function.

### A-ODN Degradation in Medium Containing Pollen Tubes

**Figure 8:**
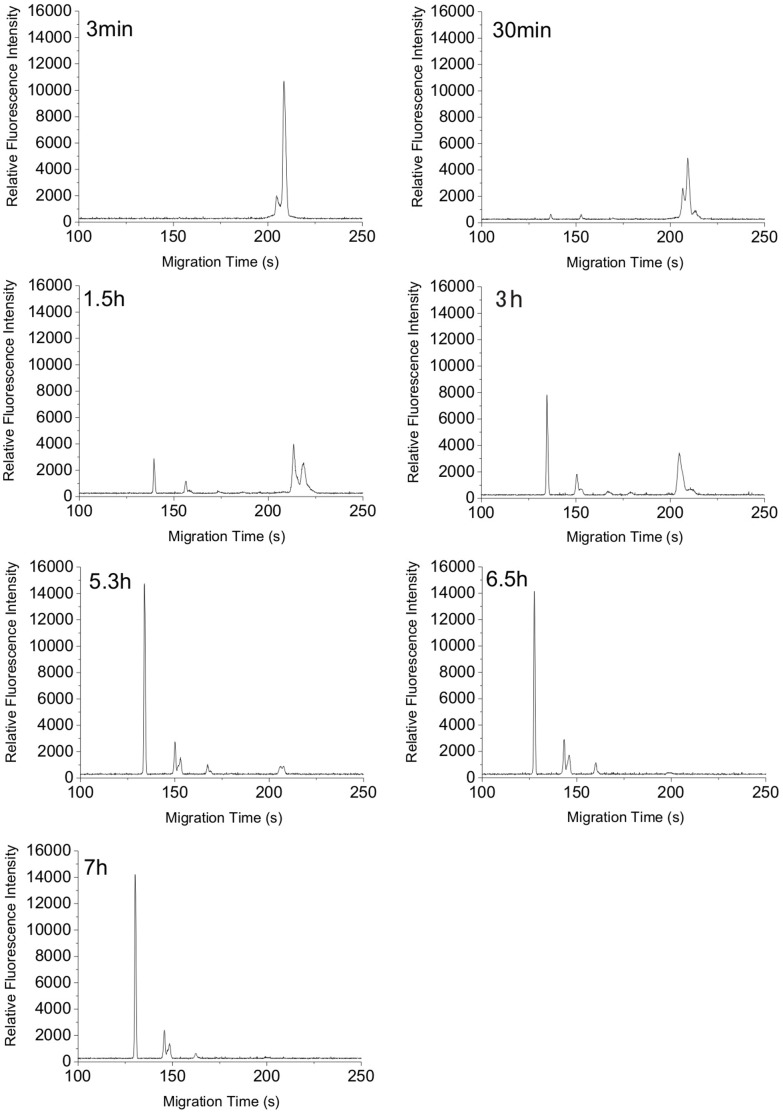
Capillary electrophoresis analysis of FL-ODN in PGM containing pollen. The migration time of FL-ODN peaks ranged from more than 200 s to less than 150 s during 7 h incubation, indicating the degradation begin within 30 mins and lasted to 7^th^ hour.

To determine why the ODNs lost their inhibitory effect as the treatment was prolonged (*e.g*., after 8 h. Fig. 8), we used capillary electrophoresis analysis to trace FL-ODN in pollen culture medium (PGM) during an 8-h treatment period. As we expect, FL-ODN in PGM degraded slowly over the 8-h period; a decrease in fluorescence became noticeable after approximately 30 min and had almost disappeared by the 7^th^ hour (Fig. 8); this was in comparison to FL-ODN in PGM with no pollen, in which FL-ODN was stable over the 8-h period (Fig. S3). These data indicate that FL-ODN may be degraded by the growing pollen tubes.

## Discussion

### The A-ODN Technique is an Efficient Assay for Gene Function Analysis in Pollen Tubes

A-ODNs offer an alternative method to disrupt normal gene expression in animals or in nucleic acid therapeutics [36]. Given that negatively charged ODNs have difficulty crossing the plasma membrane, injection or a delivery system is often required to improve the effectiveness of internalization [37]. It is more difficult for A-ODNs to pass across the plant cell wall, as shown by the very slow incorporation of naked ODNs or calcium-precipitated ODNs into maize pollen tubes [38]. Moutiho utilized A-ODN to study target genes in pollen tubes of *Agapanthus umbellatus* [18], [19]. The A-ODNs were delivered by cationic lipids, which might have a more cytosic effect than naked A-ODN [18], [19]. Successful ODN uptake and single gene function analysis have been achieved in barley, by directly submersing the cut ends of leaves in naked ODNs [16], [17]. A-ODN has also been applied successfully to study of photosynthesis-related genes in various leaves by infiltration with a syringe [12]. Our A-ODN application system offers a convenient procedure and an alternative technique for gene function analysis in pollen tubes, which is an important model for the study of polar cell growth.

In our experiments, we used a simple medium containing A-ODNs for pollen tube germination and growth. The fluorescently labeled ODNs were quickly observed in pollen tubes, indicating highly effective ODN uptake during pollen tube growth under our intended conditions. Recently, it has been found that sucrose, and active transport of mono- or disaccharides through sugar translocators, can greatly enhance ODN uptake [17]. This strategy was used to deliver ODNs into barley seeds, endosperm cells, and even human cells [17]. It was also reported that wheat is sensitive to osmotic stress; *e*.*g*., treatment with 200 mM sucrose [12]. The medium we used for pollen tube growth also contained a high concentration of sucrose (20%). Our result supports the hypothesis that the highly effective uptake of ODN is related to sugar; however, the detailed mechanism remains unclear.

The overlap of fluorescently labeled ODNs and FM4-64 at the tip of the pollen tube suggested that the peculiar membrane trafficking at the apex might also facilitate the uptake of A-ODNs. Although the detailed mechanism of ODN uptake in both cases still requires careful investigation, the successful application of the technique in pollen tubes offers a convenient alternative procedure for gene function analysis using pollen tubes as a model system.

Eliminating off-target effects is a great challenge when using ODNs in gene function analysis. In our experiments, we first compared the effects of different A-ODN (ON4 and ON6) sequences targeting nearly identical mRNAs, and observed similar phenotypes, suggesting specific binding to the same target. Furthermore, the two sequences avoided non-specific inhibition, such as G4 tracts [39], [40] or the CpG motif backbone [41]. We then used reorganized sequences of ON4 and ON6 as additional controls (the random-designed ODNs). Both controls (sense and reorganized ODNs) showed no significant effect on normal pollen tube growth, even at higher concentrations. We also checked that the mRNA level of *NtGNL1* in the A-ODN-treated pollen tubes was clearly reduced (Fig. 2) and as the concentrations of ODN increased, the mRNA level in the pollen tubes decreased accordingly (Fig. 6B). Furthermore, we compared A-ODN-treated pollen tubes with RNAi transgenic lines and found similar phenotypes in terms of pollen tube growth and detailed cytological events. Together, these data suggest the carefully designed ODNs could specifically bind to the target and down-regulate gene expression. However, to ensure the effects are specific, it is necessary to use a multi-control system.

### The Effectiveness of ODN Treatment is Concentration- and Duration-dependent

Both phenotype and gene expression level analysis revealed that ODNs functioned in a concentration-dependent manner (Fig. 6). On the one hand, to ensure effective suppression of target gene expression, sufficient amounts of A-ODNs should be used, according to the material treated. On the other hand, to avoid potentially toxic effects or inhibition on pollen tube growth, excess A-ODNs should be avoided as much as possible. Nonetheless, the nature of A-ODN function offers the possibility to create weak or strong phenotypes, according to the requirement for gene function analysis. This is especially useful when attempting to elucidate complex cytological dynamics, such as in vesicle trafficking.

Our data also revealed that A-ODN treatment was duration-dependent (Fig. 7). This was confirmed by both phenotype analysis and the A-ODN degradation analysis. In terms of the effective duration, the inhibition of A-ODN had a dynamic effect, increasing at first and decreasing later. Thus, the most effective time point may vary among plant materials and primary concentrations. A-ODN itself proved to be stable in the medium used for pollen tube growth, as indicated by the capillary electrophoresis analysis (Fig. S3). However, when ODNs were incubated with pollen tubes, degradation occurred (Fig. 8). Thus, data quality depends in part on determining the effective duration of ODN treatment. In addition, this effective duration may be variable among plant species and when different ODN sequences are used.

Regarding the mechanism of the observed A-ODN degradation, it is possible that enzymes released from pollen tubes may catalyze A-ODN breakdown and gradually render them non-functional. Interestingly, when A-ODNs were exhausted, pollen tubes recovered completely, showing normal growth. This again suggests that the toxicity associated with the A-ODNs used in these experiments was negligible; generally, pollen tubes are very sensitive to any harmful influence and in this case the growth-inhibiting effect was reversible [42], [43]. This phenomenon also suggests that the A-ODN technique may be useful for transiently interrupting gene function, allowing researchers to non-destructively observe the roles of a target gene in different or specific developmental stages. Obviously, this is not possible when using traditional RNAi or gene mutation techniques for the same purpose.

### Entry of A-ODN into Pollen Tubes

The biological effects of A-ODN have been attributed to the uptake route. Alam [21] found that A-ODNs conjugated to a bivalent RGD *vs.* unconjugated ODNs were internalized via distinct endocytic pathways. In our research, we did not conjugate A-ODNs to any other ligand (*i.e.,* so-called “naked” ODN delivery). Endocytosis in pollen tubes can involve two routes: one is bulk-phase endocytosis, which overlaps with FM4-64 dye. The other is receptor-mediated endocytosis, which happens only in the apical membrane, also traced by FM4-64. In tobacco, distinct endocytic pathways were revealed using positively or negatively charged nanogold, suggesting that clathrin-dependent endocytosis occurs in the apex and subapical regions of the pollen tube [44]. Our results (Fig. 1B) showed that FL-ODNs partially overlapped with FM4-64 staining, indicating that the entry route of naked A-ODN may not be bulk-phase endocytosis, but rather may be receptor-mediated endocytosis. Although A-ODN has a negative charge, like negatively charged nanogold, our results do not identify the endocytic pathway by which A-ODN is translocated. Entry of A-ODN has been examined in animal cells [22], but the details of this pathway remain unknown.

## Materials and Methods

### Plant Materials

*Nicotiana tabacum* cv. Petite Havana SR1 plants were grown under 16 h of daylight at 25°C in a greenhouse or axenically in incubators. Anthers were collected at room temperature to release pollen into pollen germination medium (PGM).

### Pollen Germination and Pollen Tube Growth

Pollen was cultured in PGM. The medium was modified from Sun et al. [45]: 20% (w/v) sucrose, 0.01% (w/v) boric acid, 0.1 mM calcium chloride, 3 mM methyl ester sulfonate (MES), pH 5.6, incubated in the dark at 25°C. ODNs were dissolved in PGM and then cocultured with pollen from 0 to 10 h.

### A-ODN Selection and Pollen Tube Treatment

ODN sequences were designed using principles of nucleic acid thermostability by picking several 18−20-bp antisense fragments from the mRNA of the targeted gene *NtGNL1* (Table 1), all with phosphorothioate at both the 5′- and 3′-ends. All sequences had high GC percentages (>60%) and were synthesized by Invitrogen (Carlsbad, CA, USA) or TaKaRa (Tokyo, Japan). According to preliminary experiments, ON4 was chosen for the inhibition of pollen tube growth. Both sense sequences and scrambled sequences of ON4 were designed as controls. After comparing their effects on pollen tubes cultured without ODN, we selected scrambled sequences of ON4 as controls in all related experiments (scrambled ON4∶5'-CCG TGA CCT GCA CGA CGC-3'). The ODNs were directly dissolved in PGM.

### Pollen Tube RNA Extraction and Semiquantitative RT-PCR

RNA extractions were carried out using the TRIzol reagent (Gibco-BRL, Grand Island, NY, USA). Pollen tubes were collected by centrifugation (3000 rpm); the supernatant was discarded and TRIzol was added according to the manufacturer’s instructions. RNA samples were adjusted to equal concentrations using a spectrophotometer and RNA electrophoresis. RNA reverse transcription was performed using the SuperScript II Reverse Transcriptase kit (Invitrogen). Total RNA (2 µg) was used as the template together with 1 µL oligo(dT)_12–18_ (25 µg/µL) in a final reaction volume of 20 µL. Two primers were used for amplifying *NtGNL1* (NCBI, EF520731: upstream primer rtu1∶5′-GGC ATC AGC GAC TTT GAC CAA-3′; upstream primer rtu2∶5′-GCT TCC GAT TGG TTC ATC-3′; downstream primer rtl1∶5′-CTT GTT TCT TGC CAG CCT CTG-3′; downstream primer rtl2∶5′-GTG ACT TGC CCA TGG ATT-3′). Tubulin was chosen as an internal control (tbu2∶5′- CAC CAA CCT TAA CCG CCT TA-3′; tbl2∶5′-GCT GCT CAT GGT AAG CCT TC-3′; designed from *N. tabacum* tubA2 mRNA, NCBI Accession Number AJ421412).

### Cytological and Ultrastructural Observations

We used fluorescein diacetate (FDA) dye (1 µg/mL) to check pollen tube viability after A-ODN treatment. ODNs were labeled with Alex Fluor 488 at the C terminal (TaKaRa synthesized them) for tracing the entry pathway into pollen tubes. Pollen tubes were incubated in labeled ODNs (250 nM) in medium for at least 1 h, and then mounted on slides for observation. Images were taken with a CCD or confocal microscope (Leica SP2).

After 3 h of culture, pollen tubes were used for loading FM4-64, according to a previously described method [21]. Then, the pollen tubes were centrifuged (10000 rpm) to remove the dye and were transferred to pollen germination medium for observation. The ultrastructural examination was according to Liao *et al*. [30].

### Capillary Electrophoresis (CE) Analysis

The electrophoresis buffer consisted of 20 mM sodium tetraborate (pH 9.2). A new capillary was pre-treated with 1.0 M NaOH, and water for 30 min, sequentially. Prior to use, the capillary was rinsed with 0.1 M NaOH, and water for 5 min, followed by preconditioning with running buffer for 10 min. Separations were carried out at a constant voltage of 20 kV and the operating current was 25.5−26 µA. The sample injection was performed in hydrodynamic mode with sampling height at 10 cm for 42 s. The germination media containing ODN, with or without pollen, were analyzed by CE with fluorescence detection.

Capillary electrophoresis was carried out using a laboratory-built system, based on an upright fluorescence microscope (Olympus, Japan), a photo-multiplier tube (PMT), a ±30-kV high-voltage DC power supply (Shanghai Institute of Nuclear Research, China), and an uncoated fused-silica capillary of 50 cm (28.5−29 cm length to the detector window)×50 µm I.D. ×365 µm O.D. (Yongnian Optical Conductive Fiber Plant, China), as reported previously [46], [47].

### Microscopy and Data Analysis

Microscopic observations and image collection were performed according to Wang et al. [23]. Data analysis was performed as described previously [24]. All data were analyzed using the Microsoft Excel 2000 software (*P* value, Student’s *t*-test) and independent-sample *t*-test using the SPSS (16.0) software.

## Supporting Information

Figure S1 **The efficiency of Fl-ODNs entering pollen tubes within 3 hours.** Dye number means the number of pollen tubes with Fl-ODN signals; patch-like means the number of pollen tubes, in which Fl-ODN signals were patch-like distributed and equal means the number of pollen tubes, in which Fl-ODN signals were equal-distributed. n = 260±12. The double asterisks indicate *P*<0.01, asterisk indicates *P*<0.05. The data were calculated and analyzed by SPSS (16.0) Independent-Sample T Test. Error bars in the columns represent SD.
(TIF)

Figure S2 **The test of potential toxic effect on pollen tube growth and pollen tube viability.** Control (A,B) and A-ODN4 (C, D). Both of them showed high viability. A and C are bight field images. B and D are fluorescent images. Pollen tubes were labeled by FDA. Bar = 100µm.
(TIF)

Figure S3 **Capillary electrophoresis analysis of germination medium containing ODN without pollen.** The migration time of FLODN peaks kept at near 200s during 7 hours, which means early no FL-ODN degradation occurred.
(TIF)
