# Supplementary figures and images for: Antisense Oligodeoxynucleotide Inhibition as an Alternative and Convenient Method for Gene Function Analysis in Pollen Tubes
Source: PLoS One. 2013 Mar 20;8(3):e59112. doi: 10.1371/journal.pone.0059112 (PMC3604054; doi:10.1371/journal.pone.0059112)

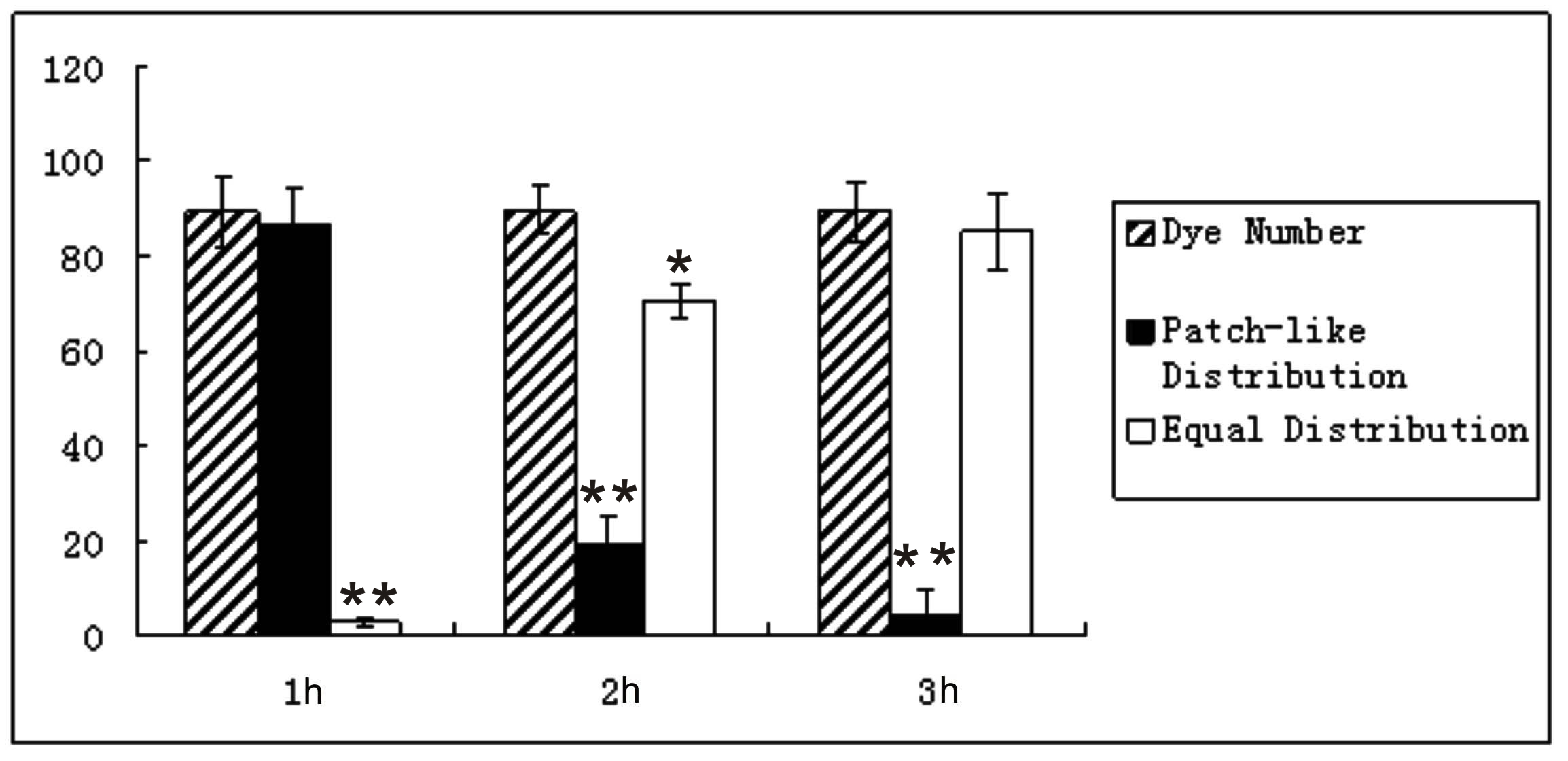

Supplement: Figure S1 — The efficiency of Fl-ODNs entering pollen tubes within 3 hours. Dye number means the number of pollen tubes with Fl-ODN signals; patch-like means the number of pollen tubes, in which Fl-ODN signals were patch-like distributed and equal means the number of pollen tubes, in which Fl-ODN signals were equal-distributed. n = 260±12. The double asterisks indicate P<0.01, asterisk indicates P<0.05. The data were calculated and analyzed by SPSS (16.0) Independent-Sample T Test. Error bars in the columns represent SD. (TIF) [file pone.0059112.s001.tif]

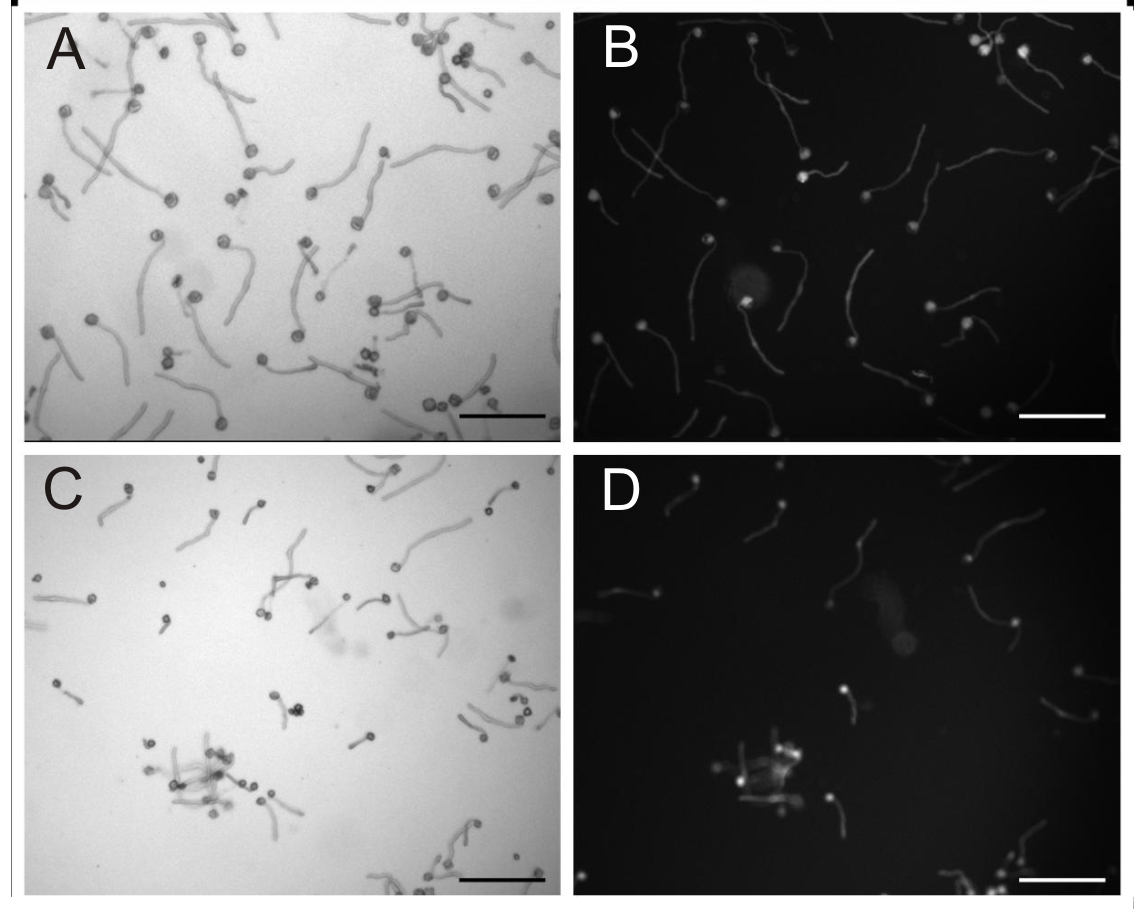

Supplement: Figure S2 — The test of potential toxic effect on pollen tube growth and pollen tube viability. Control (A,B) and A-ODN4 (C, D). Both of them showed high viability. A and C are bight field images. B and D are fluorescent images. Pollen tubes were labeled by FDA. Bar = 100µm. (TIF) [file pone.0059112.s002.tif]

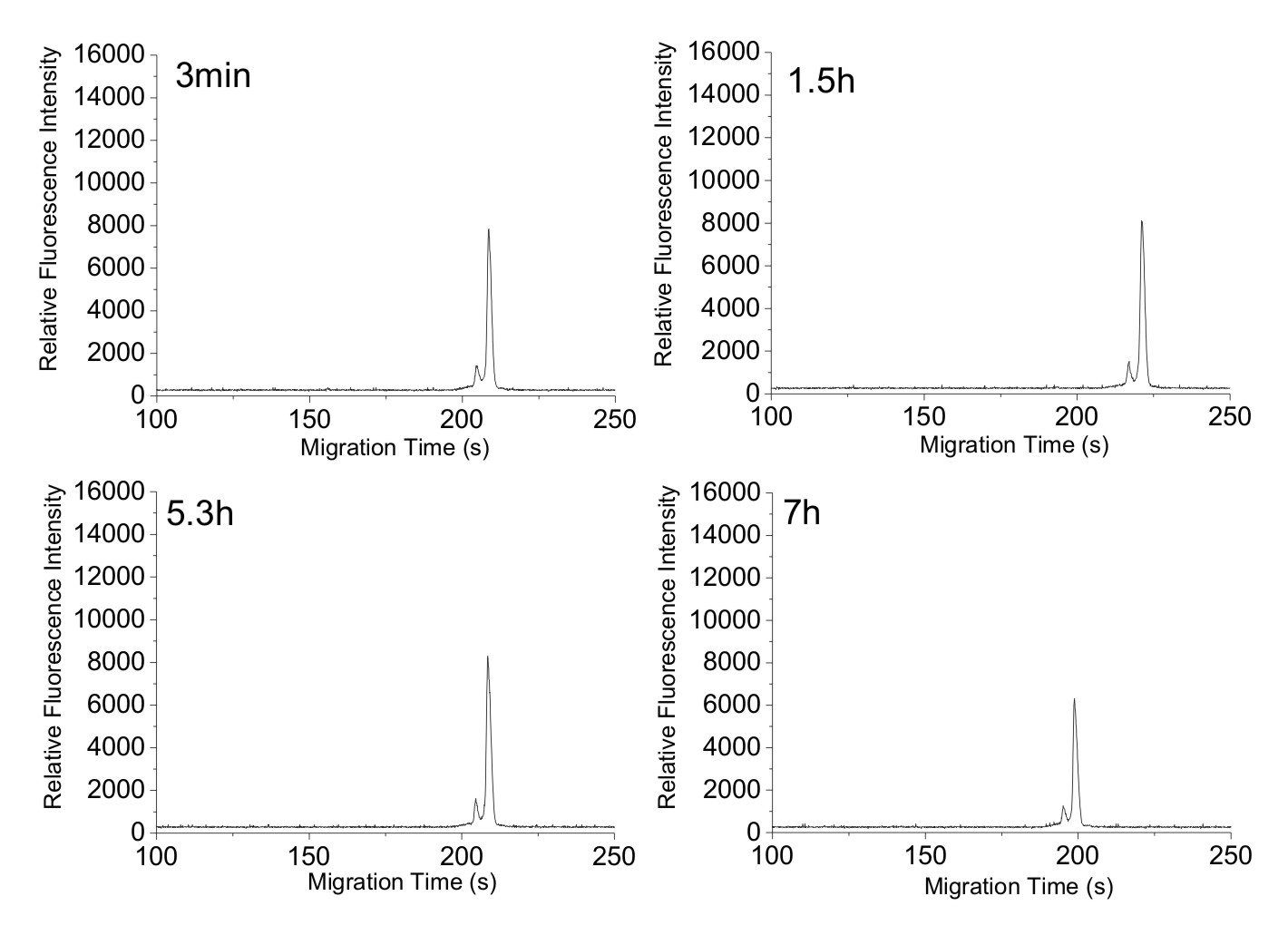

Supplement: Figure S3 — Capillary electrophoresis analysis of germination medium containing ODN without pollen. The migration time of FLODN peaks kept at near 200s during 7 hours, which means early no FL-ODN degradation occurred. (TIF) [file pone.0059112.s003.tif]
